# Prevalence of maternal mortality causes based on ICD-MM: a systematic review and meta-analysis

**DOI:** 10.1186/s12884-023-06142-y

**Published:** 2023-11-28

**Authors:** Jahanpour Alipour, Abolfazl Payandeh, Afsaneh Karimi

**Affiliations:** 1 Health Human Resources Research Center, School of Health Management & Information Sciences, Shiraz University of Medical Sciences, Shiraz, Iran; 2 Department of Biostatistics and Epidemiology, Infectious Diseases and Tropical Medicine Research Center, Research Institute of Cellular and Molecular Sciences in Infectious Diseases, Zahedan University of Medical Sciences, Zahedan, Iran; 3 Pregnancy Health Research Center, Zahedan University of Medical Sciences, Zahedan, Iran

**Keywords:** Maternal mortality, Maternal death, ICD-MM, Systematic review, Meta-analysis, Prevalence

## Abstract

**Background:**

Maternal mortality is a universal public health challenge. ICD-Maternal Mortality (ICD-MM) was introduced in 2012 to facilitate the gathering, analysis, and interpretation of data on maternal deaths worldwide. The present study aimed to estimate the global prevalence of maternal death causes through a systematic review and meta-analysis.

**Methods:**

A systematic literature search was conducted using various databases, including Web of Science, PubMed, Scopus, ScienceDirect, Cochrane Library, as well as Persian databases such as MagIran and Scientific Information Database (SID). The search encompassed articles published until August 21, 2022. Thirty-four eligible articles were included in the final analysis. Analysis was performed using a meta-analysis approach. The exact Clopper-Pearson confidence intervals, heterogeneity assessment, and random effects models with Mantel-Haenszel methods were employed using the STATA software version 14.2.

**Results:**

The most prevalent causes of maternal deaths, listed in descending order from highest to lowest prevalence, were non-obstetric complications (48.32%), obstetric hemorrhage (17.63%), hypertensive disorders of pregnancy, childbirth, and the puerperium (14.01%), other obstetric complications (7.11%), pregnancy with abortive outcome (5.41%), pregnancy-related infection (5.26%), unanticipated complications of management (2.25%), unknown/undetermined causes (2.01%), and coincidental causes (1.59%), respectively.

**Conclusion:**

Non-obstetric complications, obstetric hemorrhage, and hypertensive disorders of pregnancy, childbirth, and puerperium were the most common causes of maternal deaths. To reduce the burden of maternal mortality causes, increasing awareness and promoting self-care management among women of reproductive age, and implementing effective screening mechanisms for high-risk mothers during pregnancy, childbirth, and the puerperium can play a significant role. ICD-MM enables the uniform collection and comparison of maternal death information at different levels (local, national, and international) by facilitating the consistent collection, analysis, and interpretation of data on maternal deaths. Our findings can be utilized by policymakers and managers at various levels to facilitate necessary planning aimed at reducing the burden of maternal mortality causes.

**Supplementary Information:**

The online version contains supplementary material available at 10.1186/s12884-023-06142-y.

## Background

Accurate death statistics are important for public health decision-making and evaluating the effectiveness of public health programs. The absence of reliable data on the mortality causes can result in the provision of low-quality health services [1, 2]. Maternal mortalities are among the avoidable deaths that have garnered national, international, and political commitments for reduction. Identification of suitable strategies to prevent them is considered one of the most important programs in most countries [3–5].

According to the WHO, “A maternal death is the death of a woman while pregnant or within 42 days of termination of pregnancy, irrespective of the duration and the site of the pregnancy, from any cause related to or aggravated by the pregnancy or its management, but not from accidental or incidental causes” [6]. The maternal mortality ratio (MMR) is a key public health indicator that quantifies the number of maternal deaths per 100,000 live births. It serves as a reflection of the quality of health services and the position and importance accorded to women in the society [7–9]. The global MMR has decreased significantly from 342 in 2000 to 211 in 2017 [10]. However, despite global efforts to minimize maternal deaths, this index remains high [11]. According to the WHO statistics in 2017, approximately 810 women died every day from preventable causes related to pregnancy and childbirth, with 94% of these deaths occurring in low and lower-middle-income countries. In less developed countries, MMR is 415 per 100,000 live births, which is very high compared to Europe and North America (12 per 100,000 live births) as well as Australia and New Zealand (7 per 100,000 live births) [12–14].

Maternal health is one of the key priorities of the WHO. Furthermore, in line with the supplementary goal of Sustainable Development Goal 3 (SDG 3.1), it is targeted that no country should have a maternal mortality ratio exceeding 140 per 100,000 live births by 2030 [10, 15, 16]. To achieve this goal, there is a need for quality data, especially on mortality causes to design interventions and reduce the global burden of maternal mortality [17, 18]. According to the WHO, severe bleeding (mainly postpartum hemorrhage), infections (typically postpartum infections), hypertensive disorders of pregnancy (pre-eclampsia and eclampsia), complications associated with delivery, and unsafe abortion collectively contribute to 75% of maternal mortalities [12–14].

Accurate classification of the causes of maternal mortality is crucial for planning targeted interventions aimed at achieving the goal of ending avoidable maternal deaths. The WHO Application of ICD-10 to Deaths During Pregnancy, Childbirth, and the Puerperium: ICD-Maternal Mortality (ICD-MM) was introduced in 2012 with the aim of facilitating the gathering, analysis, and interpretation of data on maternal deaths worldwide consistently [19]. The purpose of this guideline is to promote the use of standardized guidelines for maternal death classification, aiming at enhancing usability, improving comparability of maternal deaths across local, national, and international levels, and reducing coding errors related to the cause of death (COD) [20, 21].

Given the significance of evaluating the current situation, identifying the causes, and determining related risk factors in efforts to reduce maternal mortality, this meta-analysis study was conducted with the aim of determining the common causes of maternal mortality based on ICD-MM classification. To the best of our knowledge, no previous systematic review and meta*-*analysis have comprehensively investigated maternal mortality globally using the ICD-MM classification.

## Methods

This systematic review was conducted following the Preferred Reporting item for Systematic Review and Meta-Analysis (PRISMA) Statement guideline [22] in five stages, including (1) literature review, (2) establishment of inclusion and exclusion criteria, (3) selection of relevant studies, (4) quality assessment of included studies, and (5) data analysis.

### Literature review: databases and keywords

A systematic literature search was conducted in English databases, including Web of Science, PubMed, Scopus, ScienceDirect, Cochrane Library, and Google Scholar search engines, as well as Persian databases such as MagIran and Scientific Information Database (SID), to identify relevant studies published until August 21, 2022. The following search terms were used in our search strategy: “mortality, maternal” OR “maternal mortal*” OR “maternal death*” AND “International Classification of Diseases Maternal Mortality” OR “ICD-MM”. A complementary search was conducted by manually scanning the references of the retrieved articles and previous systematic reviews on the topic to ensure all of the relevant studies were found. The details of the search strategy are presented in supplementary appendix A.

### Inclusion and exclusion criteria

The inclusion criteria for the analysis were as follows: original research studies with English abstracts, studies that focused on maternal mortality causes according to the ICD-MM classification, and studies that focused on maternal death occurring during pregnancy or within 42 days of termination of pregnancy published up to August 21, 2022.

Studies with abstracts in languages other than English, studies not reporting adequate data, studies that did not report the outcome of interest, reports retrieved from personal weblogs, systematic reviews, and letters to editors, studies that focused on maternal near miss, studies that focused on maternal death occurring after 42 days of termination of pregnancy, and ancillary or duplicate studies (studies with the same findings that have been published more than once with the same or different titles) were excluded from analysis.

### Selection of studies

At this stage, after searching the databases, the duplicated articles among the databases were removed. The remaining articles were reviewed based on the title and abstract, and irrelevant articles were excluded. After that, the full texts of the remaining articles were examined using the inclusion and exclusion criteria. During this stage, each article was independently reviewed by both authors (JA and AK), and any discrepancies were resolved through the involvement of a third author (AP).

### Quality appraisal

All included articles were assessed using the standard checklist of the Newcastle Ottawa Scale (NOS Scale) [23]. The evaluation process was carried out by two independent reviewers, and any discrepancies were resolved through discussion or consultation with a third author, if necessary. The interrater reliability was high (κ = 0.98). The eligibility of the articles in this step was determined based on the NOS score. A NOS score of 7–9 indicated high-level quality, a score of 5–6 indicated moderate-level quality, and a score below 5 was considered low-level quality. Low quality studies were excluded. Out of 29 studies, only one with a score of 5 was excluded. Of the remaining 28 studies, one study [19] with a score of 6 and moderate quality and the remaining 27 studies with a score of 7 or more and high quality were included in the meta-analysis.

### Statistical analysis

After the quality appraisal process, the full text of each study was reviewed, and the data were extracted from the studies using a predesigned form (Supplementary Appendix B). The form included basic characteristics of the studies such as the authors’ names, publication year, study design, location, country classification according to the World Bank 2023 [24], participants’ age, and sample size. Furthermore, the form contained nine maternal mortality causes based on ICD-MM. The causes included in the analysis were categorized into nine groups: six direct maternal mortality causes (1. abortive outcome, 2. hypertensive disorders in pregnancy, childbirth, and the puerperium, 3. obstetric hemorrhage, 4. pregnancy-related infection, 5. other obstetric complications, and 6. unanticipated complications), one indirect maternal mortality cause (7. non-obstetric complications), 8. unknown/undetermined causes, and 9. coincidental causes [6].

The analysis of extracted data was carried out using a meta-analysis approach. The exact Clopper–Pearson confidence intervals were computed to determine the error proportion of each individual study [25]. Heterogeneity was quantified using the Cochran Q-test (p < 0.1) and the *I*^*2*^ index. The *I*^*2*^ values of 25%, 50%, and 75% indicate low, moderate, and high heterogeneity, respectively. All analyses were conducted using a random-effects model with the Mantel-Haenszel method. Random-effects models are preferred as they are more conservative and have better properties, particularly in the presence of heterogeneity [26]. The pooled prevalence of maternal death causes and their 95% confidence intervals were computed using the *metan* command in the STATA. Any potential publication bias was explored using Begg’s test and Egger’s test, which evaluate whether the precision of the studies is related to the magnitude of their effect size. All analyses were performed using the STATA software version 14.2 and Microsoft Excel 2019.

## Results

**Fig. 1:**
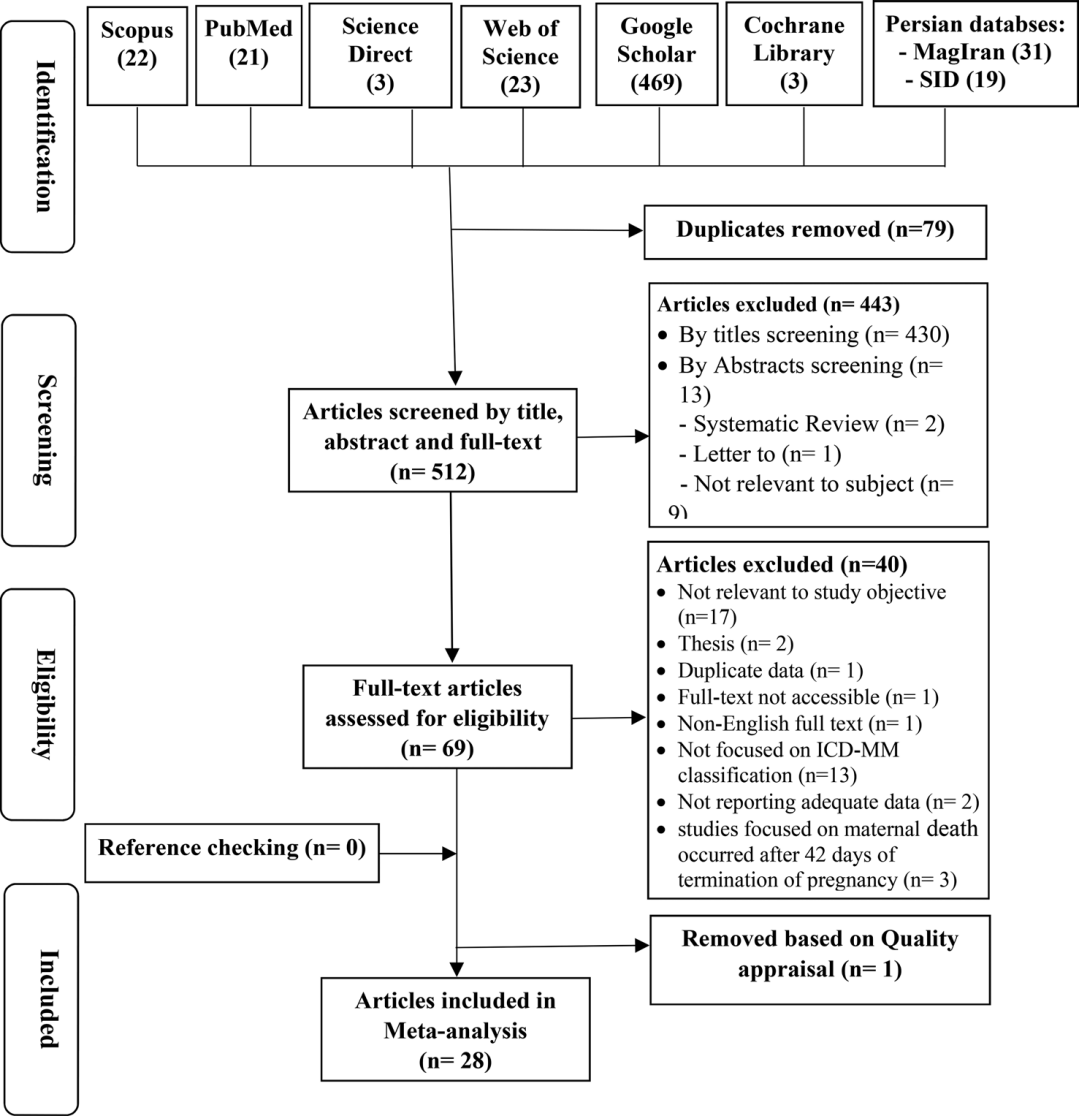
PRISMA flow diagram

The systematic search of the databases initially yielded a total of 591 articles, out of which 79 articles were identified as duplicates and subsequently removed. The remaining 512 articles underwent screening based on their titles and abstracts, resulting in the exclusion of 430 and 13 articles, respectively. The full texts of the remaining 69 articles were assessed for compliance with the inclusion and exclusion criteria, leading to the exclusion of 40 articles. The details of the selection process are presented in Fig. 1. Among the included studies, three cross-sectional studies [27–29] reported maternal mortality causes for multiple years separately. For analysis purposes, the findings of each reported year were considered as separate studies. As a result, the number of included articles in the meta-analysis increased to 34.

A total of 34 studies were found to be eligible for inclusion in the meta-analysis, consisting of 3 studies conducted in developed countries and 31 studies conducted in developing countries. The most common study designs among the included studies were cross-sectional (94%), with only two studies utilizing a cohort design.

**Fig. 2:**
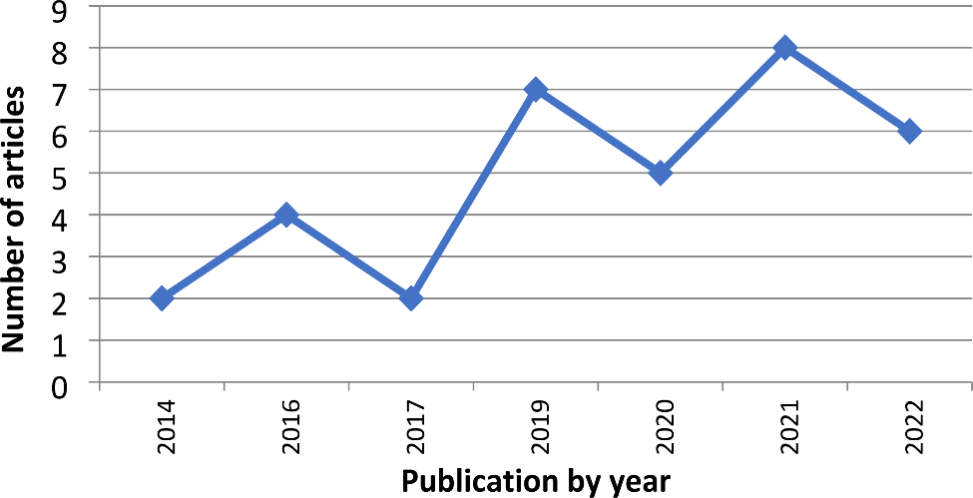
Yearly distribution of published articles

The majority of the studies were published within the last four years (n = 26,76.5%), with the highest frequency of published articles observed in 2021 (n = 8, 23.5%) (Fig. 2).

**Fig. 3:**
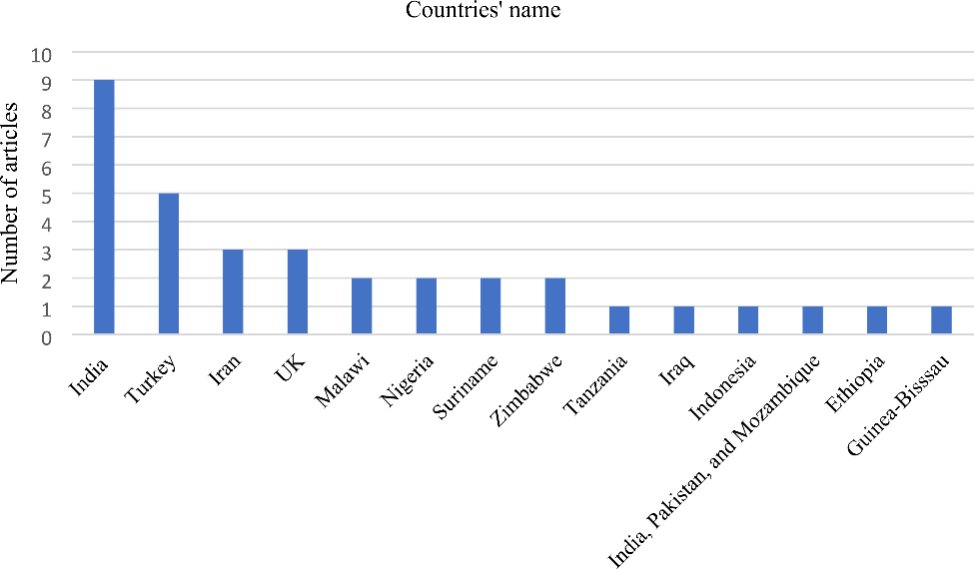
Country distribution of published articles

India had the highest number of published articles (n = 9, 26.5%), followed by Turkey (n = 5, 14.7%). Iran and the UK each had three studies, while Malawi, Nigeria, Suriname, and Zimbabwe each had two studies. The remaining countries contributed only one published study in the search field (Fig. 3).

**Table 1:** Characteristics of the subjects (34 studies)

First author	Publication year	Country	Country classification	Study design	N	Method of assigning cause of death	Reference
Halder	2014	India	Developing	Cross sectional	212	Audit team	[30]
Owolabi	2014	Malawi	Developing	Cross sectional	54	Healthcare providers using a standard MDR form	[31]
Knight	2016	UK	Developed	Cross sectional	276	Expert Panel	[27]
Knight	2016	UK	Developed	Cross sectional	269	Expert Panel	[27]
Knight	2016	UK	Developed	Cross sectional	240	Expert Panel	[27]
Mgawadere	2016	Malawi	Developing	Cross sectional	86	Expert Panel	[32]
Cim	2017	Turkey	Developing	Cross sectional	69	Maternal Mortality Review Commission	[33]
Kodan	2017	Suriname	Developing	Cross sectional	65	Expert committee	[17]
Aikpitanyi	2019	Nigeria	Developing	Cross sectional	18	Maternal and Perinatal Death Surveillance and Response Committee of the hospital	[34]
Barzegar	2019	Iran	Developing	Cross sectional	53	Expert Panel	[35]
Engin-Üstün	2019	Turkey	Developing	Cross sectional	812	Investigation Committee	[28]
Engin-Üstün	2019	Turkey	Developing	Cross sectional	812	Investigation Committee	[28]
Engin-Üstün	2019	Turkey	Developing	Cross sectional	812	Investigation Committee	[28]
Engin-Üstün	2019	Turkey	Developing	Cross sectional	812	Investigation Committee	[28]
Sheth	2019	India	Developing	Cross sectional	295	Trained Consultants	[19]
Agrawal	2020	India	Developing	Cross sectional	382	Not Stated	[36]
Kumari	2020	India	Developing	Cohort	131	Maternal mortality review committee	[37]
Mohammed	2020	Iraq	Developing	Cross sectional	206	Registry-based	[38]
Said	2020	Tanzania	Developing	Cross sectional	99	Expert Panel	[39]
Sharma	2020	India	Developing	Cross sectional	42	Maternal death review committee	[40]
Akaba	2021	Nigeria	Developing	Cross sectional	52	Expert Panel	[41]
Aukes	2021	India, Pakistan, and Mozambique	Developing	Cohort	143	Expert reviewers (three doctors)	[42]
Ekka	2021	India	Developing	Cross sectional	142	The facility maternal death review committee and the Institutional death review committee	[43]
Indarti	2021	Indonesia	Developing	Cross sectional	22	MDSR forms	[44]
Jogi	2021	India	Developing	Cross sectional	112	Facility-Based Maternal Death Review Form and MDR Case Summary	[45]
Karimi	2021	Iran	Developing	Cross-sectional	107	MDR committee of hospital	[5]
Kodan	2021	Suriname	Developing	Cross-sectional	73	National MDR committee	[46]
Sarmalkar	2021	India	Developing	Cross sectional	305	Not Stated	[47]
Aghajani	2022	Iran	Developing	Cross-sectional	80	Expert panel	[48]
Kea	2022	Ethiopia	Developing	Cross-sectional	48	MDSR forms	[49]
Musarandega	2022	Zimbabwe	Developing	Cross-sectional	325	Trained obstetricians	[29]
Musarandega	2022	Zimbabwe	Developing	Cross-sectional	137	Trained obstetricians	[29]
Nóbrega	2022	Guinea-Bissau	Developing	Cross sectional	79	Registry-based (EU/PIMI-II)	[16]
Verma	2022	India	Developing	Cross sectional	135	Not Stated	[7]

A summary of the included studies, including the first author, publication year, country, country classification, study design, and sample size, is presented in Table 1. Approximately 91% of the studies were conducted in developing countries.

**Table 2:** Frequency of maternal mortality causes of study subjects (34 studies)

First author	Year	Country	N	Maternal mortality causes N (%)									Reference
				1. Pregnancy with abortive outcome	2. Hypertensive disorders in pregnancy, childbirth and the puerperium	3. Obstetric hemorrhage	4. Pregnancy-related infection	5. Other obstetric complications	6. Unanticipated complications of management	7. non-obstetric complications	8. Unknown/undetermined causes of death	9. Coincidental causes	
Halder	2014	India	212	14 (6.6)	26 (12.6)	25 (11.8)	27 (12.7)	3 (1.4)	4 (1.9)	102 (48.1)	4 (1.9)	7 (3.3)	[30]
Owolabi	2014	Malawi	54	2 (3.8)	3 (5.7)	17 (32.1)	12 (22.6)	1 (1.9)	0 (0)	13 (24.5)	3 (5.7)	1 (1.9)	[31]
Knight	2016	UK	276	4 (1.4)	10 (3.6)	14 (5.1)	16 (5.8)	43 (15.6)	3) 1.1)	163 (59.1)	0 (0)	23 (8.3)	[27]
Knight	2016	UK	269	8 (3)	9 (3.3)	11 (4.1)	13 (4.8)	44 (16.4)	4 (1.5)	154 (57.2)	0 (0)	26 (9.7)	[27]
Knight	2016	UK	240	6 (2.5)	6 (2.5)	13 (5.4)	8 (3.3)	47 (19.6)	3 (1.3)	131 (54.6)	0 (0)	26 (10.8)	[27]
Mgawadere	2016	Malawi	86	12(14)	10 (1.6)	29 (33.7)	12 (14)	2 (2.3)	1 (1.2)	15 (17.4)	5 (5.8)	0 (0)	[32]
Cim	2017	Turkey	69	0 (0)	18 (45)	11 (27.5)	0 (0)	11 (17.5)	0 (0)	17 (24.6)	0 (0)	12	[33]
Kodan	2017	Suriname	65	2 (3)	9 (14)	13 (20)	6 (9)	11 (17)	0 (0)	21 (32)	3 (5)	0 (0)	[17]
Aikpitanyi	2019	Nigeria	18	0 (0)	1 (5.6)	12 (66.6)	2 (11.1)	3 (16.7)	0 (0)	0 (0)	0 (0)	0 (0)	[34]
Barzegar	2019	Iran	53	3 (5.7)	7 (13.2)	12 (22.6)	0 (0)	7 (13.2)	0 (0)	24 (45.3)	0 (0)	0 (0)	[35]
Engin-Üstün	2019	Turkey	812	0 (0)	33 (17.2)	35 (18.2)	5 (2.6)	40 (20.9)	0 (0)	77 (40.1)	2 (1)	0 (0)	[28]
Engin-Üstün	2019	Turkey	812	0 (0)	38 (17)	42 (18.8)	9 (4)	23 (10.3)	0 (0)	107 (47.8)	5 (2.2)	0 (0)	[28]
Engin-Üstün	2019	Turkey	812	0 (0)	29 (13.6)	41 (19.2)	14 (6.6)	29 (13.6)	0 (0)	94 (44.3)	6 (2.8)	0 (0)	[28]
Engin-Üstün	2019	Turkey	812	0 (0)	26 (14.2)	28 (151.3)	4 (2.2)	19 (10.4)	0 (0)	94 (51.5)	12 (6.6)	0 (0)	[28]
Sheth	2019	India	295	7 (2.4)	55 (18.6)	71 (241.1)	20 (6.8)	14 (4.7)	6 (2)	105 (35.6)	16 (5.4)	1 (0.3)	[19]
Agrawal	2020	India	382	17 (4.4)	46 (11.9)	42 (11)	20 (5.6)	28 (7.2)	0 (0)	229 (60)	0 (0)	-	[36]
Kumari	2020	India	131	8 (6.1)	41 (31.2)	25 (19)	19 (14.5)	1 (0.8)	0 (0)	37 (28.2 )	0 (0)	0 (0)	[37]
Mohammed	2020	Iraq	206	7 (3.4)	16 (7.8)	65 (31.6)	4 (1.9)	25 (12.1)	4 (1.9)	42 (20.4)	43 (20.9)	-	[38]
Said	2020	Tanzania	99	7 (7.1)	16 (16.2)	38 (38.4)	8 (8.1)	11 (11.1)	6 (6.1)	11 (11.1)	1 (1)	1 (1)	[39]
Sharma	2020	India	42	-	9 (21.4)	5 (11.9)	7 (16.6)	0 (0)	3 (7.1)	18 (42.9)	0 (0)	-	[40]
Akaba	2021	Nigeria	52	4 (7.7)	19 (36.5)	16 (30.7)	9 (17.3)	0 (0)	1 (1.9)	3(5.7)	0 (0)	0 (0)	[41]
Aukes	2021	India, Pakistan, and Mozambique	143	1 (1)	23 (16)	38 (27)	6 (4)	16 (11)	4 (3)	39 (27)	16 (11)	0 (0)	[42]
Ekka	2021	India	142	4 (2.8)	51 (35.9)	37 (26.1)	21 (14.8)	2 (1.4)	2 (1.4)	25 (17.6)	-	-	[43]
Indarti	2021	Indonesia	22	0 (0)	2 (9.1)	2 (9.1)	1 (4.5)	1 (4.5)	3 (13.6)	11 (50)	0 (0)	2 (9.1)	[44]
Jogi	2021	India	112	2 (1.8)	41 (36.6)	29 (25.9)	16 (14.3)	2 (1.8)	2 (1.8)	20 (17.9)	0 (0)	-	[45]
Karimi	2021	Iran	107	2 (1.9)	18 (16.8)	22 (20.6)	12 (11.2)	24 (22.4)	1(0.9)	22 (20.6)	3 (2.8)	3 (2.8)	[5]
Kodan	2021	Suriname	73	2 (3)	7 (10)	13 (18)	6 (8)	13 (18)	0 (0)	21 (29)	3 (4)	8 (11)	[46]
Sarmalkar	2021	India	305	15 (4.9)	42 (13.7)	45 (14.8)	11 (3.6)	9 (2.9)	0 (0)	182 (59.7)	1 (0.3)	-	[47]
Aghajani	2022	Iran	80	0 (0)	9 (11.2)	14 (17.5)	10 (12.5)	4 (5)	2 (2.5)	41 (51.2)	0 (0)	-	[48]
Kea	2022	Ethiopia	48	3 (6)	10 (21)	21 (44)	1 (2)	8 (17)	0 (0)	5 (8)	-	-	[49]
Musarandega	2022	Zimbabwe	325	30 (9.2)	34 (10.5)	64 (19.7)	33 (10.1)	14 (4.3)	5 (1.5)	113 (34.8)	19 (5.8)	13 (4)	[29]
Musarandega	2022	Zimbabwe	137	24 (17.5)	25 (18.2)	34 (24.8)	7 (5.1)	12 (8.7)	1 (0.7)	29 (21.2)	3 (2.2)	2 (1.4)	[29]
Nóbrega	2022	Guinea-Bissau	79	0 (0)	10 (12.6)	35 (44.3)	0 (0)	0 (0)	7 (8.9)	23 (29.1)	4 (5)	-	[16]
Verma	2022	India	135	0 (0)	29 (25.2)	30 (26)	18 (15.7)	5 (4.3)	0 (0)	33 (28.7)	0 (0)	0	[7]

Table 2 presents the frequency of maternal mortality causes reported in each study.

**Table 3:** Results of meta-analysis (Random-effects model)

Causes group	Maternal mortality Cause	N^*^	Sample Size	Prevalence (%)	95% CI	P	Q	${\varvec{I}}^{2}$ (%)
7	Non-obstetric complications	33	7487	48.32	(48.24, 48.39)	< 0.001	5.20000	100.0
3	Obstetric hemorrhage	34	7505	17.63	(17.58, 17.70)	< 0.001	35249.60	99.9
2	Hypertensive disorders in pregnancy, childbirth and the puerperium	34	7505	14.01	(13.96, 14.07)	< 0.001	24716.39	99.9
5	Other obstetric complications	34	7505	7.11	(7.07, 7.15)	< 0.001	160,000	100.0
1	Pregnancy with abortive outcome	33	7463	5.41	(3.83, 6.98)	< 0.001	23604.34	99.9
4	Pregnancy-related infection	34	7505	5.26	(5.23, 5.30)	< 0.001	33.678.07	99.9
6	Unanticipated complications of management	34	7505	2.25	(2.22, 2.29)	< 0.001	2468.02	99.4
8	Unknown/undetermined causes of death	32	7315	2.01	(1.99, 2.04)	< 0.001	29262.25	100
9	Coincidental causes	25	6151	1.59	(1.55, 1.62)	< 0.001	66974.44	100.0

*N = Number of studies

Heterogeneity statistics indicated a high level of heterogeneity among the included studies (p *<* 0.001). Hence, random-effects models were applied to all studies for each type of error. Begg’s and Egger’s test also indicated that there was no publication bias in any of the studies regarding all types of maternal mortality causes (P > 0.05). The pooled prevalence of maternal mortality causes and their 95% confidence intervals are reported in Table 3. This table also indicates the number of eligible studies and the total number of subjects in each cause-specific meta-analysis. Overall, nine meta-analyses were performed. Forest plots were eliminated because of setting limitations and redundant reporting of findings in the paper. According to Table 3, non-obstetric complications were found to be the most prevalent cause of maternal mortality, while coincidental causes were identified as the least common cause.

## Discussion

This meta-analysis included 34 studies and aimed to pool the prevalence of maternal mortality causes worldwide. The majority of the included studies (91%) were conducted in developing countries, with 76% of them published within the last four years. Notably, more than a quarter of the studies were conducted in India.

Our findings revealed that non-obstetric complications were the most prevalent cause of maternal mortality in the included studies. Except for a study conducted by Aikpitanyi et al. [34] who examined but did not report any cases of non-obstetric complications leading to maternal deaths, the remaining 33 studies reported different rates ranging from 5 to 60% for this group of causes. Knight et al. [49] reported a higher average rate of non-maternal deaths in the UK compared to developing countries. Previous studies [50, 51] have also highlighted that in developed countries, indirect causes of maternal mortality, particularly non-obstetric complications, are more prevalent than direct causes. This has been attributed to the well-organized antenatal care programs in these countries.

Obstetric hemorrhages were identified as the second most prevalent cause among all causes and the first most common cause among direct causes of maternal deaths in the included articles. All of the studies examined this group of causes and reported rates ranging from 4.1 to 66.6%. About 62% of maternal mortalities worldwide can be attributed to direct causes, with obstetric hemorrhages accounting for nearly half of them (about 27%) [50]. Postpartum hemorrhages are the most common cause of obstetric hemorrhages (72%) [51] accounting for about 25% of maternal deaths globally [52]. This is despite the fact that the majority of maternal deaths caused by hemorrhages are preventable [53, 54]. It has been estimated that, on average, uterine contraction is responsible for 75% of postpartum hemorrhages [55]. Therefore, the control and treatment of uterine contraction play a significant role in reducing the incidence of maternal deaths.

The findings of the present study revealed that hypertensive disorders in pregnancy, childbirth, and the puerperium were the third most prevalent cause of maternal mortality among all causes and the second most prevalent cause among direct causes (ranging from 2.5 to 45%). These findings are in line with the global prevalence of hypertensive disorders in pregnancy reported as the second most common cause of maternal mortality [56]. Similar to our findings, Keskinkılıç et al. [57] reported that hypertensive disorders were the third most prevalent cause among all causes and the second most common cause among direct causes of maternal deaths in Turkey. Hypertensive disorders of pregnancy include pre-existing (chronic) or pregnancy-induced hypertension and are strongly associated with cardiac attack and stroke [56]. Screening and identifying pregnant women with hypertensive disorders, providing appropriate treatment, and enhancing their self-care management through mobile-based applications can help control the disease and reduce mortality associated with these conditions.

We found that other obstetric complications were the fourth most prevalent cause of maternal mortality (7.11%) that ranged from 1.4% in India to 22.4% in Iran. This group included “blood clot embolism, amniotic fluid embolism, intentional self-harm (death by suicide), complications of obstetric surgery and procedures (such as disruption of cesarean section wound, cardiomyopathy, and other complications of the puerperium)” [58]. On average, the risk of venous thromboembolism is 4.5 times higher in pregnant women compared to non-pregnant women. Furthermore, advanced maternal age (over 35) and underlying conditions such as thrombophilia, lupus erythematosus, cardiovascular disease, postpartum infections, and obesity have been identified as serious risk factors for thromboembolism [59]. Rath and Stelzl [60] found that appropriate pharmacological thromboprophylaxis could reduce the risk of venous thrombosis by about 65%. Developing and implementing effective monitoring mechanisms for high-risk mothers and designing standard care, treatment, and surgery methods for pregnant patients can reduce the rate of maternal mortality due to this group of causes.

Pregnancy with abortive outcome was the fifth most prevalent cause (5.41%, ranging from 1 to 17.5%) among all causes and the fourth cause among direct causes. Pregnancy with abortive outcome includes “abortion, miscarriage, ectopic pregnancy, and other conditions leading to maternal death and a pregnancy with abortive outcome” [61]. Unsafe abortion is one of the most common causes of maternal mortality across the world, with higher rates in countries with stricter abortion laws [62]. Abortion law reforms in countries with restricted abortion, policy-making and planning for prevention and early detection of ectopic pregnancy risk factors, and promoting the awareness of women of reproductive age can reduce maternal mortalities due to these conditions.

The present study found that pregnancy-related infection was the sixth most prevalent maternal mortality cause among all causes (5.26%, ranging from 1.9 to 22.6%) in the reviewed studies. Obstetric infections are widely recognized as a significant contributor to maternal mortality worldwide, with a higher incidence observed in low- and middle-income countries. [63]. Pregnancy-related infections include “endometritis, peritonitis, pelvic abscess, surgical site infection, and necrotizing fasciitis.” [61] Improving the timeliness and accuracy of management for obstetric infections, ensuring access to appropriate healthcare facilities, and addressing delays in seeking care are crucial factors in reducing maternal mortality associated with pregnancy-related infections [64]. Therefore, promoting early diagnosis, facilitating timely access to proper care, and ensuring effective treatment can significantly reduce the burden of obstetric infections.

Unanticipated complications of management were the seventh most common cause of maternal mortality (2.25%, ranging from 1.1 to 13.6%). This group of maternal mortality causes includes conditions such as “severe adverse defects and other unanticipated complications of medical and surgical care during pregnancy, childbirth, or the puerperium” [61]. Despite the low prevalence of this group of maternal mortality causes, analysis of its prevalence provides valuable information that can shed light on the epidemic of iatrogenic-induced maternal mortality.

The present study revealed that unknown/undetermined maternal death was the eighth most prevalent cause of maternal mortality (2.01%, ranging from 0.3 to 20.9%) in the included studies. This group of causes refers to maternal deaths that occur during pregnancy, childbirth, or the puerperium, but the underlying cause of death is either unknown or cannot be determined [61]. Coincidental causes were identified as the least prevalent (ninth most common) causes of maternal mortality (1.9%, ranging from 0.3 to 11%). Coincidental causes are the deaths that occur during pregnancy, childbirth, or the puerperium due to external causes [61]. Coincidental causes are likely to be constant during and after pregnancy because they are not caused by pregnancy. However, being pregnant at the time of such conditions can increase the risk of maternal death.

The present meta-analysis analyzed the prevalence of causes of maternal deaths based on the international standard (ICD-MM) globally. Therefore, the results of the study can have the potential to encourage researchers and healthcare managers at different levels (local, national, and international) to collect, analyze, interpret, and compare the causes of maternal deaths using this international standard.

### Study limitation

The present study had some limitations including (1) several factors such as selected databases, keywords, and processes to search for studies, inclusion and exclusion criteria of studies and the number of samples included in the meta-analysis can affect the results of the meta-analysis of the prevalence of maternal causes of deaths. Therefore, it is conceivable that other similar studies with modifications to each of the previously mentioned factors will result in partially different results. (2) The results of the present study focused on the prevalence of maternal deaths reported based on the ICD-MM classification, so many studies that investigated the causes of maternal deaths based on other sources were excluded. (3) The present meta-analysis excluded maternal near misses to focus solely on the maternal deaths that occurred. Including this item in similar studies may result in different results from the present study. (4) The difference in the process of determining the causes of maternal death in the included studies is another limitation of this study and this may negatively affect the reliability of the results of this meta-analysis. and (5) The heterogeneity among included studies was considerable in our meta-analysis. There were several possible sources of heterogeneity, including differences in the method of assigning the cause of death, the study design, the sample size, or the country classification. We employed the random-effects model technique to reduce the amount of heterogeneity.

## Conclusion

The most prevalent causes of maternal mortality, in descending order, were non-obstetric complications, obstetric hemorrhage, hypertensive disorders in pregnancy, childbirth, and the puerperium, other obstetric complications, pregnancy with abortive outcome, pregnancy-related infection, unanticipated complications of management, unknown/undetermined causes, and coincidental causes. Accurate and timely diagnosis and treatment of conditions complicating pregnancy, childbirth, and the puerperium continue to pose a significant gap in high-quality care for pregnant women worldwide, particularly in developing countries where the incidence is higher.

To reduce the burden of maternal mortality causes, it is crucial to implement effective screening mechanisms for high-risk mothers during pregnancy, childbirth, and the puerperium and apply standard treatment methods. Additionally, increasing awareness and promoting self-care management among women of reproductive age can play a significant role in this regard. Our findings offer comprehensive and standardized information on the prevalence of maternal mortality causes using the ICD-MM classification. This information can be utilized by policymakers and managers at various levels to facilitate necessary planning aimed at reducing the burden of maternal mortality causes.

### Electronic supplementary material

Below is the link to the electronic supplementary material.

**Supplementary Material 1**: Appendix A - Search Strategy

**Supplementary Material 2**: Appendix B - Data Extraction Form

## Data Availability

All data analysed in this study are included within the article and list of references.

## Abbreviations

ICD-MM: The WHO Application of ICD-10 to Deaths During Pregnancy, Childbirth and Puerperium
SID: Scientific Information Database
STATA: Statistical Software for Data Sciences
WHO: World Health Organization
MMR: Maternal Mortality Ratio
ICD-10: International Classification of Disease
COD: Cause of Death
PRISMA: The Preferred Reporting Items for Systematic Reviews and Meta-Analysis
