# Supplementary material for: Prevalence of maternal mortality causes based on ICD-MM: a systematic review and meta-analysis
Source: BMC Pregnancy Childbirth. 2023 Nov 28;23:821. doi: 10.1186/s12884-023-06142-y (PMC10683107; doi:10.1186/s12884-023-06142-y)
Supplement: Supplementary file 1 — Supplementary Material 1: Appendix A - Search Strategy [file 12884_2023_6142_MOESM1_ESM.docx]

Supplementary Appendix A- The details of the search strategy

Search date: August 21, 2022

| Database | Search # | Query | Results |
| --- | --- | --- | --- |
| Web of Science | #1 | **"mortality, maternal"** (All Fields) or **"maternal mortal*"** (All Fields) or **"maternal death*"** (All Fields) | [16,552](https://www2.wosgs.ir/wos/woscc/summary/1177ab05-2f2d-4b77-98a1-2054f8b65655-499dd49f/relevance/1) |
|  | #2 | **(ALL=("International Classification of Diseases Maternal Mortality")) OR ALL=("ICD-MM" )** | [23](https://www2.wosgs.ir/wos/woscc/summary/575b156c-7037-4d88-a4c5-8100e4ca1312-499de0b7/relevance/1) |
|  | #3 | **#1 AND #2** | [23](https://www2.wosgs.ir/wos/woscc/summary/5de218ec-68c1-4e09-b146-ae4687f1a7c0-499df0d8/relevance/1) |
|  | #4 | #1 AND #2 and English (Languages) | [22](https://www2.wosgs.ir/wos/woscc/summary/8ce4f6e0-d6c8-4807-b0d6-3d9b65109414-499dfff5/relevance/1) |
| PubMed | #1 | "mortality, maternal"[All Fields] OR "maternal mortal*"[All Fields] OR "maternal death*"[All Fields] | 20458 |
|  | #2 | "International Classification of Diseases Maternal Mortality"[All Fields] OR "international classification of diseases maternal mortality"[All Fields] OR "ICD-MM"[All Fields] | 21 |
|  | #3 | #1 AND #2 | 21 |
|  | #4 | #1 AND #2 AND (english[Filter]) | 20 |
| Scopus | #1 | ( TITLE-ABS-KEY ( "mortality, maternal" ) OR TITLE-ABS-KEY ( "maternal mortal*" ) OR TITLE-ABS-KEY ( "maternal death*" ) ) | [34,252](https://www.scopus.com/search/history/results.uri?origin=searchhistory&shid=10) |
|  | #2 | ( TITLE-ABS-KEY ( "International Classification of Diseases Maternal Mortality" ) OR TITLE-ABS-KEY ( "ICD-MM" ) ) | [22](https://www.scopus.com/search/history/results.uri?origin=searchhistory&shid=6) |
|  | #3 | #1 AND #2 | [22](https://www.scopus.com/search/history/results.uri?origin=searchhistory&shid=6) |
|  | #4 | #1 AND #2 AND  ( LIMIT-TO ( LANGUAGE ,  "English" ) ) | [20](https://www.scopus.com/search/history/results.uri?origin=searchhistory&shid=11) |
| Science Direct | #1 | Title, abstract, keywords: "mortality, maternal" OR "maternal mortality" OR "maternal death" OR "maternal mortalities" | 4,959 |
|  | #2 | Title, abstract, keywords: "International Classification of Diseases Maternal Mortality" OR "ICD-MM" | [4](https://www2.wosgs.ir/wos/woscc/summary/575b156c-7037-4d88-a4c5-8100e4ca1312-499de0b7/relevance/1) |
|  | #3 | **#1 AND #2**  Title, abstract, keywords: ("mortality, maternal" OR "maternal mortality" OR "maternal death" OR "maternal moralities") AND ("International Classification of Diseases Maternal Mortality" OR "ICD-MM") | [3](https://www2.wosgs.ir/wos/woscc/summary/5de218ec-68c1-4e09-b146-ae4687f1a7c0-499df0d8/relevance/1) |
| Cochrane Library | #1 | ("mortality, maternal"):ti,ab,kw OR ("maternal mortal*"):ti,ab,kw OR ("maternal death*"):ti,ab,kw | 1205 |
|  | #2 | ("International Classification of Diseases Maternal Mortality"):ti,ab,kw OR ("ICD-MM"):ti,ab,kw | [3](https://www2.wosgs.ir/wos/woscc/summary/575b156c-7037-4d88-a4c5-8100e4ca1312-499de0b7/relevance/1) |
|  | #3 | **#1 AND #2** | [3](https://www2.wosgs.ir/wos/woscc/summary/5de218ec-68c1-4e09-b146-ae4687f1a7c0-499df0d8/relevance/1) |
| Google scholar | #1 | allintext:"maternal mortality" OR "maternal mortalities" OR "maternal death" | 18200 |
|  | #2 | allintext:"ICD-MM"OR "International Classification of Diseases Maternal Mortality" | 606 |
|  | #3 | **#1 AND #2**  allintext:"maternal mortality" OR "maternal mortalities" OR "maternal death" "ICD-MM"OR "International Classification of Diseases Maternal Mortality" | 469 |
| MagIran | #1 | "maternal mortality" OR "maternal mortalities" OR "maternal death" | 31 |
| SID | #1 | "maternal mortality" OR "maternal mortalities" OR "maternal death" | 19 |
